# Cdk4 Regulates Recruitment of Quiescent β-Cells and Ductal Epithelial Progenitors to Reconstitute β-Cell Mass

**DOI:** 10.1371/journal.pone.0008653

**Published:** 2010-01-13

**Authors:** Ji-Hyeon Lee, Junghyo Jo, Anandwardhan A. Hardikar, Vipul Periwal, Sushil G. Rane

**Affiliations:** 1 Regenerative Biology Section, Diabetes Branch, National Institute of Diabetes and Digestive and Kidney Diseases, National Institutes of Health, Bethesda, Maryland, United States of America; 2 Laboratory of Biological Modeling, National Institute of Diabetes and Digestive and Kidney Diseases, National Institutes of Health, Bethesda, Maryland, United States of America; 3 Stem Cells and Diabetes Section, National Centre for Cell Science, Pune, Maharashtra, India; University of Bremen, Germany

## Abstract

Insulin-producing pancreatic islet β cells (β-cells) are destroyed, severely depleted or functionally impaired in diabetes. Therefore, replacing functional β-cell mass would advance clinical diabetes management. We have previously demonstrated the importance of Cdk4 in regulating β-cell mass. Cdk4-deficient mice display β-cell hypoplasia and develop diabetes, whereas β-cell hyperplasia is observed in mice expressing an active Cdk4R24C kinase. While β-cell replication appears to be the primary mechanism responsible for β-cell mass increase, considerable evidence also supports a contribution from the pancreatic ductal epithelium in generation of new β-cells. Further, while it is believed that majority of β-cells are in a state of ‘dormancy’, it is unclear if and to what extent the quiescent cells can be coaxed to participate in the β-cell regenerative response. Here, we address these queries using a model of partial pancreatectomy (PX) in Cdk4 mutant mice. To investigate the kinetics of the regeneration process precisely, we performed DNA analog-based lineage-tracing studies followed by mathematical modeling. Within a week after PX, we observed considerable proliferation of islet β-cells and ductal epithelial cells. Interestingly, the mathematical model showed that recruitment of quiescent cells into the active cell cycle promotes β-cell mass reconstitution in the Cdk4R24C pancreas. Moreover, within 24–48 hours post-PX, ductal epithelial cells expressing the transcription factor Pdx-1 dramatically increased. We also detected insulin-positive cells in the ductal epithelium along with a significant increase of islet-like cell clusters in the Cdk4R24C pancreas. We conclude that Cdk4 not only promotes β-cell replication, but also facilitates the activation of β-cell progenitors in the ductal epithelium. In addition, we show that Cdk4 controls β-cell mass by recruiting quiescent cells to enter the cell cycle. Comparing the contribution of cell proliferation and islet-like clusters to the total increase in insulin-positive cells suggests a hitherto uncharacterized large non-proliferative contribution.

## Introduction

Pancreatic β-cells are uniquely endowed with the ability to synthesize and secrete insulin – a hormone essential for glucose control [1]. Autoimmune destruction of β-cells results in Type 1 diabetes. Type 2 diabetes is characterized by significantly reduced β-cell mass that combines with β-cell dysfunction resulting in a deficit in β-cell compensation mechanisms in the face of glucose intolerance and insulin resistance [2], [3], [4]. Therefore, restoration of β-cell mass is of major clinical significance in both forms of diabetes. It is known that adult β-cells exhibit limited proliferation capacity that is dependent on genetic background [5], [6], [7]. Furthermore, β-cells turn over slowly and their proliferation potential decreases with age [8], [9]. Several potential mechanisms for regulating β-cell mass have been supported by ongoing research [10]. Pancreatic stem cells, embryonic or arising from diverse locations such as pancreatic ducts, islets and bone marrow, have been proposed as sources of insulin-producing β-cells [11], [12], [13], [14], [15], [16]. Other reported sources are trans-differentiation of pancreatic acinar cells, liver cells, differentiation of intra-islet precursors or splenocytes, and epithelial-mesenchymal transition [17], [18], [19], [20], [21], [22], [23], although recent studies have challenged some of these findings [24], [25], [26]. Furthermore, induced genetic reprogramming of adult exocrine cells to functional β-cells has been recently reported [27]. Among these possible sources, elegant lineage tracing analyses and other approaches convincingly demonstrate that β-cell self-duplication is a dominant source of adult β-cells [28], [29], [30]. A recent report shows the existence of facultative stem cells in the pancreatic ductal epithelium and their recruitment in response to an acute pancreatic injury [31]. These results suggest that the two major mechanisms that increase β-cell mass are (i) duplication of pre-existing β-cells and (ii) generation of β-cells via recruitment of facultative stem/progenitor cells within the pancreatic ductal epithelium.

The cell cycle machinery receives signals transduced by various growth factor pathways and controls cellular quiescence, proliferation, differentiation, senescence, and apoptosis [32], [33]. The retinoblastoma protein (RB) negatively regulates the passage of cells from G1 to S phase primarily by sequestering E2F transcription factors and chromatin modifiers critical for the G1/S transition. Cyclin-dependent kinases (Cdk's) promote S-phase progression and mitosis by phosphorylating and, thereby, inactivating RB. The activity of Cdk's is negatively regulated by the Ink4 and Cip/Kip families of cyclin-dependent kinase inhibitors (Cki's). Using mice with genetically modified *Cdk4* loci, we have previously shown that Cdk4 regulates β-cell mass [33], [34], [35], [36]. *Cdk4* ^−/−^ mice exhibit β-cell hypoplasia and develop diabetes, whereas *Cdk4* ^R24C/R24C^ mice (*Cdk4* ^R/R^ mice), inheriting the p16^Ink4a^-insensitive Cdk4^R24C^ kinase, exhibit β-cell hyperplasia. Several recent studies have underscored the important roles of other cell cycle regulators in β-cell biology [37], [38], [39], [40], [41], [42], [43].

While it is known that Cdk4 influences the size of the post-natal β-cell compartment, it is unknown whether this kinase regulates β-cell mass in response to regenerative stimuli and physiological demand. Cdk4^R24C^ promotes autoreactivity in the immune system when backcrossed onto the non-obese diabetic (NOD) background but it also protects pancreatic β-cell mass from autoimmune destruction when introduced into the NOD/SCID mouse [44]. While β-cell replication appears to be the primary mechanism responsible for β-cell mass increase [28], [29], [30], considerable evidence also supports a contribution from the pancreatic ductal epithelium in generation of new β-cells [12], [31], [45], [46], [47], [48]. However, the molecular regulators that govern the processes of β-cell replication or progenitor cell activation are not known and it is unclear whether Cdk4 regulates β-cell mass via either of these mechanisms. Further, while it is believed that the majority of β-cells are in a state of ‘dormancy’, it is not clear if and to what extent these quiescent cells can be coaxed to participate in the β-cell regenerative response. We address these issues in the present study. Our results demonstrate that Cdk4 enhances β-cell replication within islets and activates progenitors within the pancreatic ductal epithelium in response to partial pancreatectomy. Further, mathematical modeling of data derived upon DNA analog-based lineage-tracing suggests that Cdk4 recruits quiescent islet β-cells and ductal β-cell progenitors into a proliferation cycle. These results underscore the critical role played by Cdk4 in the regulation of β-cell mass.

## Results

### Efficient β-Cell Regeneration in *Cdk4* ^R/R^ Mice in Response to Pancreatectomy

**Figure 1:**
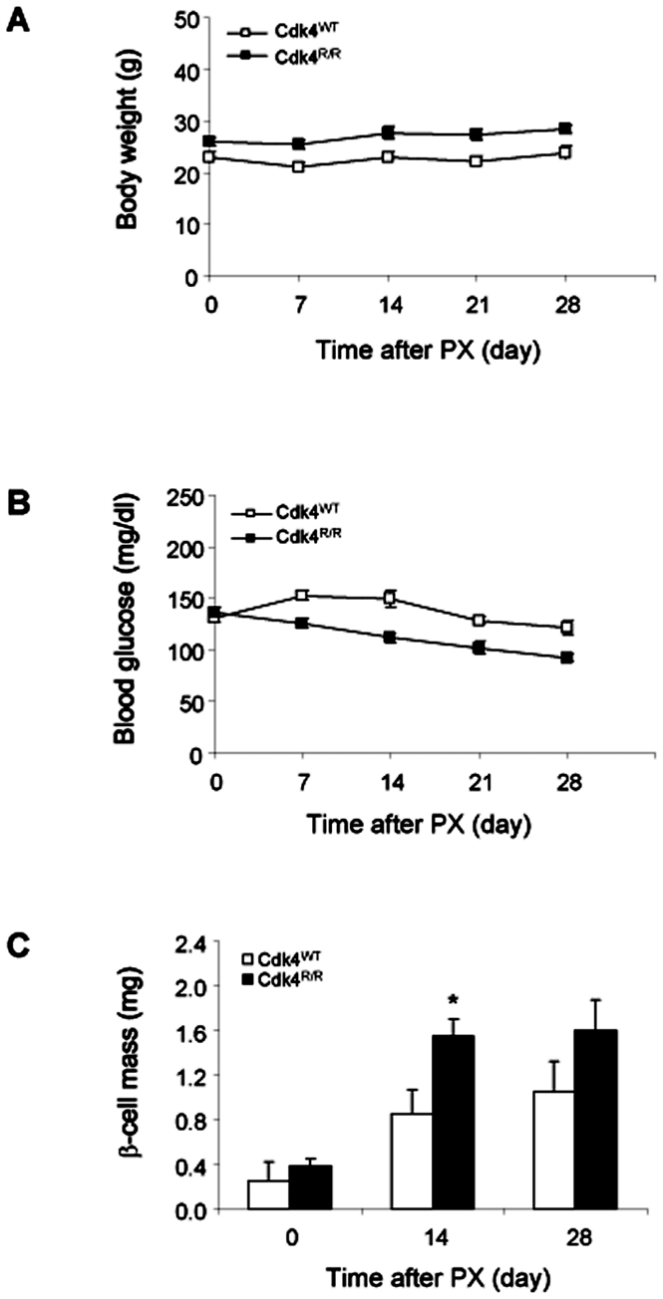
Enhanced regeneration of β-cell mass in *Cdk4* ^R/R^ mice in response to partial pancreatectomy (PX). (A) Body weight change and (B) fed-state blood glucose levels were measured before (day 0) and on indicated days after PX on *Cdk4* ^WT^ mice (open squares) and *Cdk4* ^R/R^ mice (closed squares). (C) β-cell mass is increased after PX in both groups. Significant increase of β-cell mass was observed in *Cdk4* ^R/R^ mice (closed bars) compared with *Cdk4* ^WT^ mice (open bars) by 14 days after PX. Data are shown as means and error bars represent S.E. *p<0.05 *vs Cdk4* ^WT^ mice.

Considering the critical role of Cdk4 in regulating post-natal β-cell mass [33], [36], we set out to determine its importance in controlling β-cell replication within islets and in modulating the activity of presumptive progenitor cells in the pancreatic ductal epithelium. The processes of islet β-cell replication and ductal progenitor cell activation can be simultaneously analyzed in a 60% partial pancreatectomy (PX) model [49]. We performed 60% PX in 8-week-old *Cdk4*-wild type (*Cdk4* ^WT^) and *Cdk4* ^R/R^ mice. Sham-operated mice from both genotypes served as controls. Mice were observed for a period of 28 days post-PX. No significant changes in body weights were observed in either the *Cdk4* ^WT^ or *Cdk4* ^R/R^ mice post-PX (Fig. 1A), and, consistent with prior reports [49], [50], no significant hyperglycemia was observed in either genotype (Fig. 1B), thus excluding hyperglycemia as a major factor underlying the regenerative response. Comparable β-cell mass was observed in *Cdk4* ^WT^ and *Cdk4* ^R/R^ mice immediately after PX (day 0), whereas, 14 days after PX, a significant increase in β-cell mass was observed in the *Cdk4* ^R/R^ mice, compared with *Cdk4* ^WT^ mice (Fig. 1C). The β-cell mass increase observed in *Cdk4* ^WT^ mice is consistent with that reported by Peshavaria *et al*., using a similar 60% PX model [49]. No further increase in β-cell mass was seen at day 28 post-PX in mice of either genotype. These observations indicate that Cdk4^R24C^ promotes rapid β-cell regeneration in response to PX.

### Increased Islet β-Cell Replication in *Cdk4* ^R/R^ Mice following Pancreatectomy

**Figure 2:**
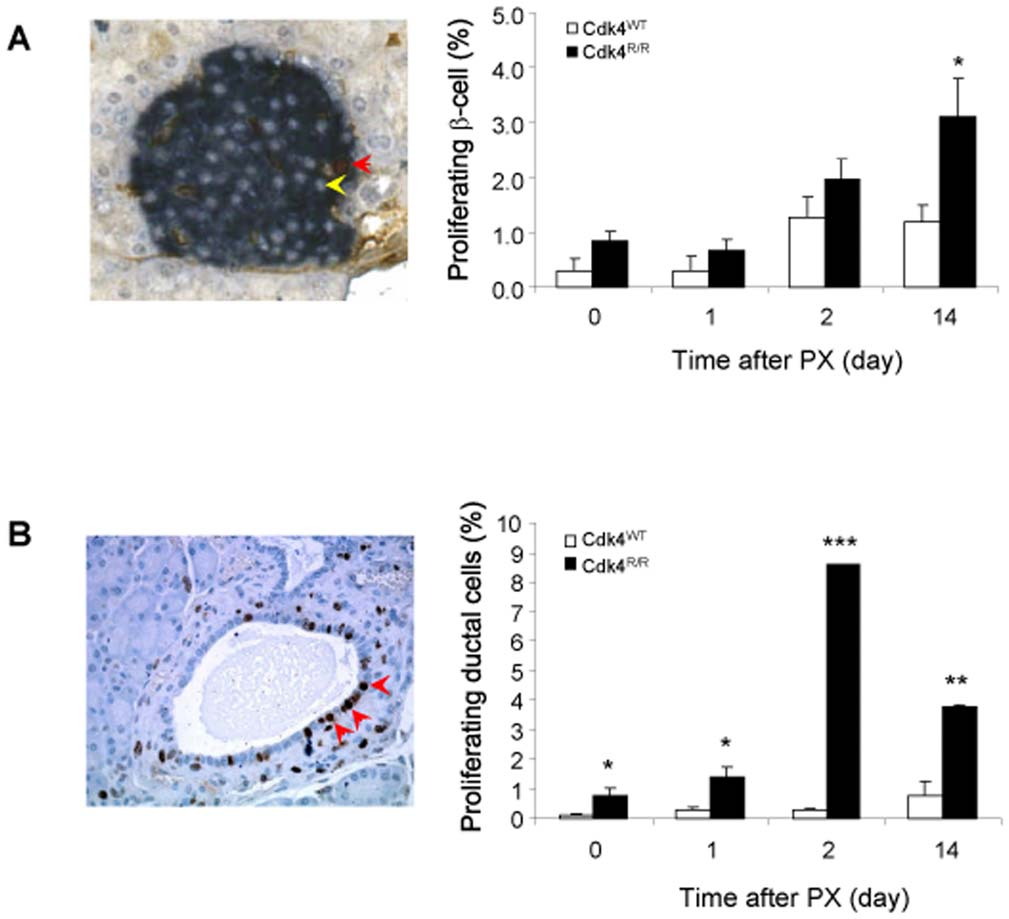
Increased islet β-cell and ductal epithelial cell proliferation after PX in *Cdk4* ^R/R^ mice. (A) Islet β-cell proliferation was significantly increased in *Cdk4* ^R/R^ mice (closed bars) compared with *Cdk4* ^WT^ mice (open bars) by 14 days after PX. The picture on the left shows BrdU^+^ (brown) and insulin^+^ (blue) β-cell (red arrow) and BrdU negative β-cell (yellow arrow). (B) Proliferation of pancreatic ductal epithelial cells was significantly increased within 2 days post-PX in *Cdk4* ^R/R^ mice (closed bars) compared with *Cdk4* ^WT^ mice (open bars). Arrow heads in picture on left indicate BrdU^+^ ductal cells. Data are shown as means and error bars represent S.E. *p<0.05, **p<0.01, and ***p<0.001 *vs Cdk4* ^WT^ mice. Note that BrdU-labeled cells result from a 16-hr-labeling period.

We quantified β-cell proliferation in the *Cdk4* ^WT^ and *Cdk4* ^R/R^ mice post-PX by staining for both bromodeoxyuridine (BrdU) and insulin (Ins) using standard immunohistochemistry (Fig. 2A). No significant difference in β-cell proliferation, as measured by counting BrdU^+^:Ins^+^ cells, was observed in *Cdk4* ^WT^ and *Cdk4* ^R/R^ pancreas immediately after PX (day 0) or 1 day after PX (Fig. 2A). A more than two-fold increase in β-cell proliferation was seen in *Cdk4* ^WT^ and *Cdk4* ^R/R^ mice by day 2 post-PX. No further increase in β-cell replication was seen in the *Cdk4* ^WT^ islets after day 2 post-PX. In contrast, a significant increase in β-cell proliferation was seen at day 14 post-PX in the *Cdk4* ^R/R^ islets. These observations demonstrate enhanced islet β-cell proliferation post-PX in *Cdk4* ^R/R^ mice.

### Enhanced Pancreatic Ductal Epithelial Cell Proliferation in *Cdk4* ^R/R^ Mice

The pancreatic ductal epithelium has been proposed to serve as a reservoir of potential facultative progenitor cells capable of differentiating into β-cells [45]. Indeed, the recruitment of such progenitors is observed in response to pancreatic injury [31]. We looked for evidence suggestive of ductal progenitor cell activation in *Cdk4* ^WT^ and *Cdk4* ^R/R^ mouse pancreas post-PX. First, we estimated pancreatic ductal epithelial cell proliferation by BrdU staining combined with staining for cytokeratin (CK) as a ductal epithelium marker. We observed very low levels of ductal cell proliferation in *Cdk4* ^WT^ pancreas prior to and at any time after PX (Fig. 2B). On the other hand, ductal cell proliferation was significantly increased in *Cdk4* ^R/R^ pancreas before PX (day 0) and at day 1 post-PX (Fig. 2B). Interestingly, ductal cell proliferation increased more than 10-fold in the *Cdk4* ^R/R^ pancreas by day 2 post-PX. The significant elevation of ductal epithelial cell proliferation was maintained in the *Cdk4* ^R/R^ pancreas until day 14 post-PX, although the absolute cell proliferation was less than that seen at day 2 post-PX. These data demonstrate robust ductal cell proliferation in the *Cdk4* ^R/R^ pancreas as early as 2 days after PX.

### Kinetics of Proliferation in Islet β-Cells and Ductal Epithelial Cells after Pancreatectomy

**Figure 3:**
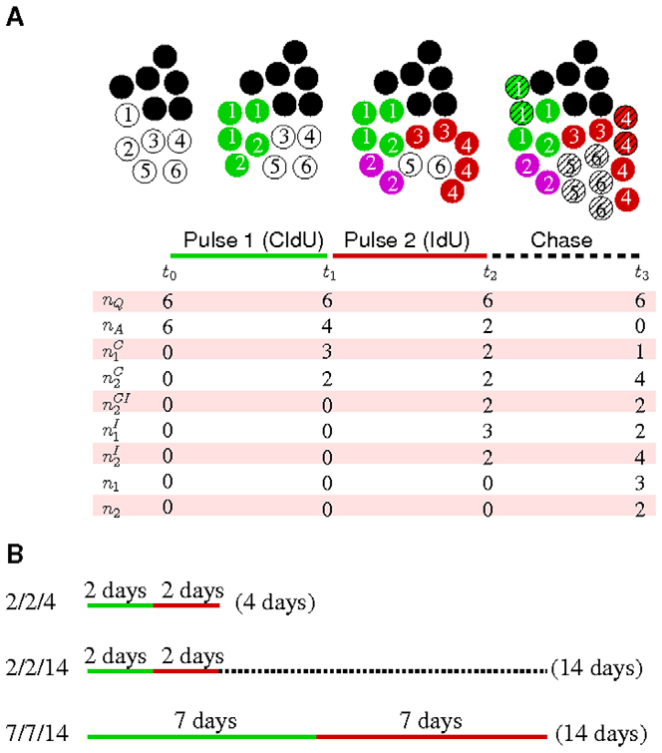
Schematic diagram and protocols of the double-labeling experiment. (A) Schematic description of cell proliferation under the double-labeling experiment. Two initial populations of 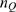 quiescent (black) and 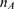 active (white) cells are subjected to two pulse (CldU and IdU) and one chase periods. The numbers of once- and twice-proliferated cells are represented by 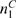 and 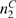 (during the CldU period); 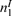 and 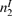 (during the IdU period); and 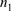 and 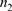 (during the chase period). The number of purple twice-proliferated (once in CldU and once in IdU) cells are denoted with 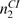. Note that some labeled cells divide during the chase period and decrease their labeling intensity. They are described with slashed green and red cells in the chase period. (B) Three protocols of the double-labeling experiment with two pulse periods and following chase period.

To observe cell-division kinetics in detail, we adapted a DNA analog-based lineage-tracing technique using dual thymidine analogs, 5-chloro-2-deoxyuridine (CldU) and 5-iodo-2-deoxyuridine (IdU) [30]. Each thymidine analog detects a distinct round of cell division; thus, it allows estimation of more than one round of cell division *in vivo* when the two analogs are sequentially provided in drinking water over defined time-periods. As schematically depicted in Fig. 3A, mice were provided CldU-containing water during the first pulse period, IdU-containing water during the second pulse period, and water without any DNA analogs during a chase period. We used three protocols each with different administration periods (Fig. 3B). The first protocol, 2-2-4 (first pulse period-second pulse period-measure time point), was designed to identify cell division kinetics within the first 4 days post-PX where, in the BrdU-labeling experiment, we had observed limited β-cell replication (Fig. 2A) but significantly enhanced ductal cell proliferation (Fig. 2B). The second protocol, 2-2-14, incorporated a 10-day chase period subsequent to the 4-day pulse period to monitor the re-division of cells that proliferated in the prior pulse period. The third protocol, 7-7-14, was designed to identify cells undergoing slow division over the entire 14 days post-PX. Immunohistochemistry with insulin and CK identified β-cells and ductal epithelium cells, respectively, and showed CldU^+^ or IdU^+^ single positive, or CldU^+^:IdU^+^ double positive cells after the labeling experiment (Figs. S1 and S2). We have summarized the labeling ratios for the three protocols in Table S1. The ratios represent accumulated cell proliferation during each labeling period.

### Mechanism of Enhanced Cell Proliferation in *Cdk4* ^R/R^ Mice

**Figure 4:**
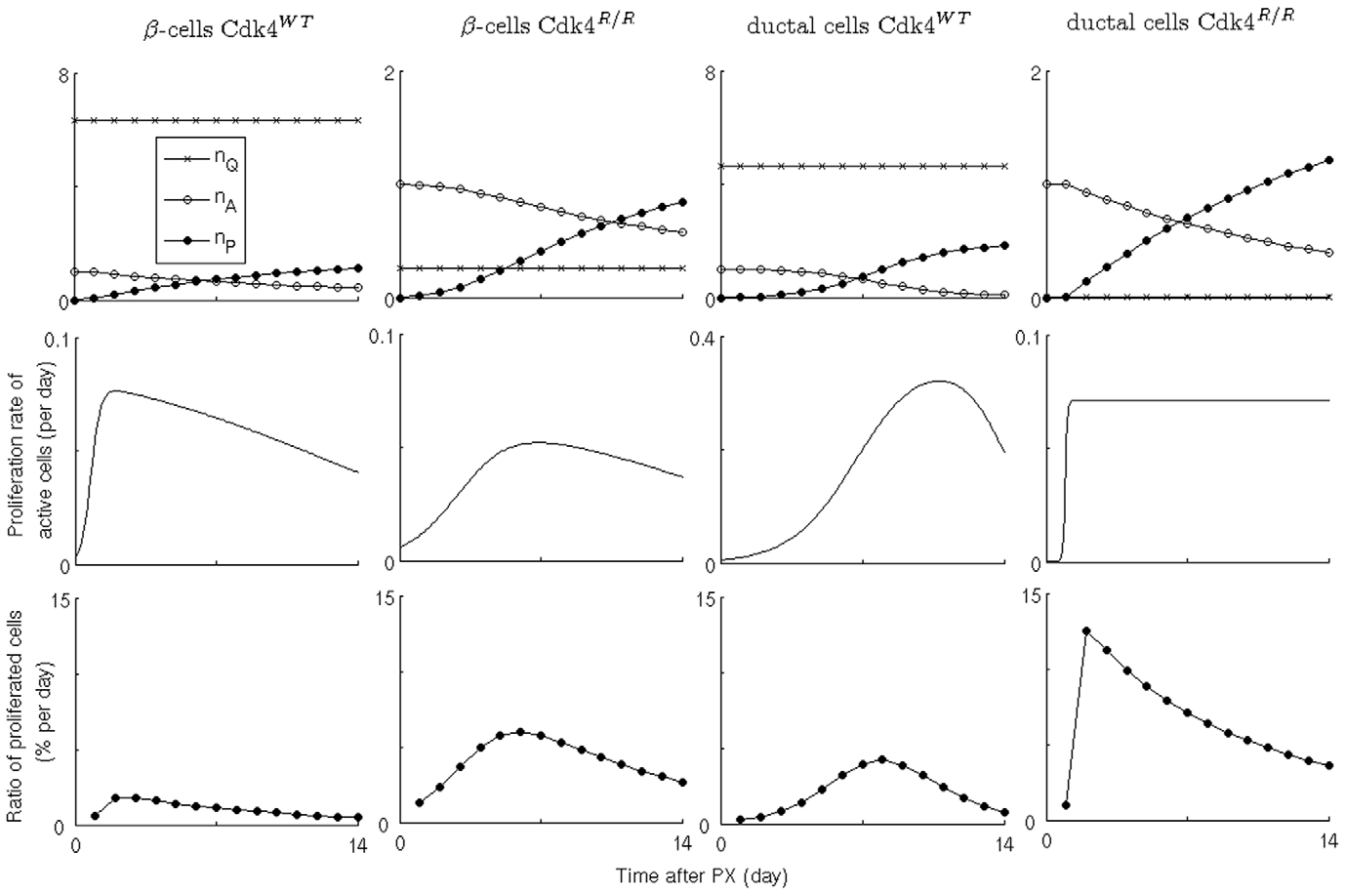
Proliferation of β-cells and ductal epithelial cells of *Cdk4* ^WT^ and *Cdk4* ^R/R^ mice after pancreatectomy. Changes of quiescent, active, and proliferated cell numbers (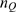,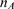, and 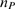) normalized by initial active cell number; proliferation rate of active cells; and ratio of proliferated cells among entire cell population are plotted.

The double-labeling experiment allows us to examine the dynamics of cell proliferation. A given labeling ratio of cell proliferation may arise from two distinct scenarios: either all cells proliferate at a certain rate or subsets of cells proliferate at a higher rate. If double-labeled cells are frequently observed, the second scenario is more probable. In this study, however, we observed rare double-labeled (CldU^+^:IdU^+^) cells within a short labeling period (14 days) regardless of genotype (Table S1). A low rate of cell division can lead to the observation of these rare double-labeled cells. However, if cells that have proliferated once require a refractory period before they undergo additional rounds of proliferation [30], despite the existence of limited highly-proliferating cells, the numbers of double-labeled cells may still remain low. We addressed these possibilities by introducing a mathematical model that allows study of the aforementioned events in quantitative detail to elucidate the relative likelihoods of these distinct scenarios (see the Materials and Methods section). In the model, we considered two cell populations of active and quiescent cells; only active cells can proliferate. In addition, we neglected multiple proliferation events of the active cells because such events are sufficiently rare. Then, we fitted the data to the models with different initial number ratios of quiescent to active cells, 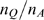. For example, 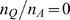 means there are no quiescent cells, i.e., every cell exhibits an equal proliferation potential. Using the cell proliferation information from the BrdU and CldU/IdU labeling experiments, the model predicted number changes of quiescent, active, and proliferated cells in islets and ducts of *Cdk4^WT^* and *Cdk4^R/R^* mice (Fig. 4); the comparison between model prediction and experimental data was shown in Table 1. Furthermore, Figure 4 showed the proliferation rate of active cells and the proportion of proliferated cells per day. Two possibilities for enhanced cell proliferation in *Cdk4^R/R^* mice are accelerated proliferation of active cells and recruitment of quiescent cells into active cell population. Our results demonstrated no difference in cell proliferation rate of active cell populations; average proliferation rates of active cells during 14 days after pancreatectomy are 16.1±12.3 vs. 16.4±12.2 (% per day) for β-cells in *Cdk4^WT^* and *Cdk4^R/R^* mice; and 15.3±10.8 vs. 7.5±0.4 (% per day) for ductal cells in *Cdk4^WT^* and *Cdk4^R/R^* mice. On the other hand, the ratio of quiescent to active cell populations decreased in both β-cells and ductal cells in *Cdk4^R/R^* mice: 48±27 vs. 18±20 for β-cells in *Cdk4^WT^* and *Cdk4^R/R^* mice; and 56±26 vs. 0±0 (0.04±0.04) for ductal cells in *Cdk4^WT^* and *Cdk4^R/R^* mice. Furthermore, model comparison, using the Bayesian model probability for the given data, 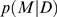, showed that a smaller quiescent cell population (small 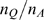) is a more likely explanation of the labeling data of *Cdk4^R/R^* mice, compared with the labeling data of *Cdk4^WT^* mice (Fig. S3).

**Table 1:** Measured and model-predicted labeling ratios.

		*Cdk4* ^WT^ β-cells		*Cdk4* ^R/R^ β-cells		*Cdk4* ^WT^ duct cells		*Cdk4* ^R/R^ duct cells	
Protocol	Labeling	Measure	Model	Measure	Model	Measure	Model	Measure	Model
Day 0	BrdU^+^	0.5±0.5	0.0	1.3±0.6	0.5	0.2±0.1	0.1	1.3±0.6	0.0
Day 1	BrdU^+^	0.5±0.5	0.6	1.1±0.4	1.3	0.4±0.4	0.3	2.8±1.0	1.0
Day 2	BrdU^+^	1.9±1.2	1.8	2.9±1.3	2.3	0.4±0.1	0.5	12.9±0.1	12.5
Day 14	BrdU^+^	1.8±0.8	1.4	4.6±1.8	2.0	1.2±1.2	1.3	5.6±0.1	2.1
2-2-4	CldU^+^	6.6±2.8	2.4	3.2±0.5	3.5	8.0±7.8	0.8	7.9±1.7	12.1
	IdU^+^	14.8±12.3	3.3	10.1±3.5	8.6	11.9±4.3	2.3	47.6±43.0	20.6
2-2-14	CldU^+^	4.6±4.3	2.3	11.2±7.3	2.8	5.5±1.8	0.6	11.7±8.8	9.0
	IdU^+^	8.9±8.4	3.2	23.0±11.3	6.8	5.6±2.7	2.0	9.0±0.7	15.3
7-7-14	CldU^+^	8.0±2.8	9.2	19.8±12.0	24.4	11.9±3.5	11.1	19.5±14.8	43.6
	IdU^+^	9.8±7.4	5.0	26.1±4.2	25.8	25.8±18.2	16.7	7.5±5.1	31.7

Numbers are average percentages of labeled cells per protocol (n = 2 to 4). The protocol name of CldU/IdU experiment represents CldU-drinking period – IdU-drinking period – measuring day. To match the scale of BrdU-labeling experiment and CldU/IdU-labeling experiment, we scale the CldU/IdU-labeling results up with 3-fold that was an optimized value (see the Materials and Methods). Raw counting numbers of labeled cells are also given in Table S1. The predicted values are the maximum likelihood values.

### Increased Pdx-1^+^ Cells in the *Cdk4* ^R/R^ Pancreatic Ductal Epithelium after Pancreatectomy

**Figure 5:**
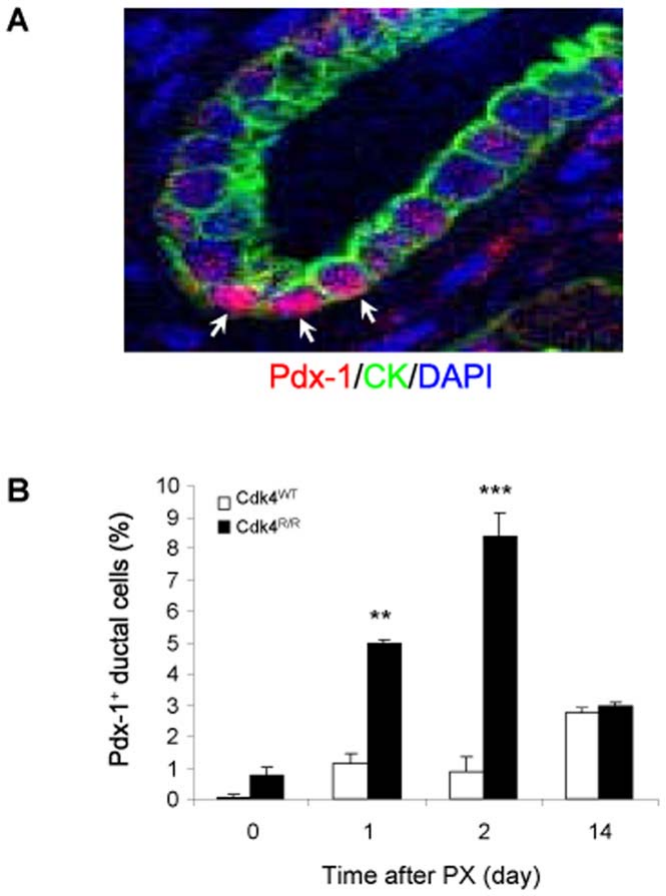
Pdx-1 expression in pancreatic ductal epithelial cells from *Cdk4* ^R/R^ mice after pancreatectomy. (A) A representative picture of Pdx-1 expression observed within the cytokeratin (CK)-positive pancreatic ductal epithelium from *Cdk4* ^R/R^ mice. Arrows show Pdx-1^+^:CK^+^ cells. Blue color identifies DAPI-stained nuclei. (B) Percentage of Pdx-1^+^ ductal epithelial cells was significantly increased by 2 days post-PX in *Cdk4* ^R/R^ mice (closed bars) compared with *Cdk4* ^WT^ mice (open bars). Data are shown as means from four mice of each genotype per group and error bars represent S.E. **p<0.01, and ***p<0.001 *vs Cdk4* ^WT^ mice.

The pancreatic duodenal homeobox 1 (Pdx-1/Idx-1/Ipf-1/Stf-1) transcription factor is an essential regulator of early pancreatic development [51], [52]. Pdx-1 is expressed in the mouse primitive gut as early as the 13 somite stage (E8.5), about a day before endocrine gene expression and 1.5 days before the first morphologic evidence of pancreas organogenesis. Subsequently, Pdx-1 is transiently present in the fetal duodenum and in pancreatic exocrine, endocrine and ductal cells, although Pdx-1 expression is suppressed in late fetal development (E15.5). In adult pancreas, Pdx-1 is localized mainly within β-cells where it functions as a transcriptional regulator of β-cell specific genes including *insulin*, *glucokinase* and *glut2*. Interestingly, factors that determine organogenesis, such as Pdx-1, frequently get activated during the regeneration processes and recapitulate stages traversed during embryonic development. Indeed, presence of Pdx-1-positive (Pdx-1^+^) cells in the pancreatic ductal epithelium has been demonstrated in rodent models following PX [12], [47], [49]. Therefore, we performed immunofluorescence assays to detect Pdx-1 expression in the pancreatic sections from *Cdk4* ^WT^ and *Cdk4* ^R/R^ mice before and after PX. The presence of ductal cells was verified by expression of CK and the number of Pdx-1 and CK double positive cells within the pancreatic ductal epithelium was determined (Fig. 5A). Prior to PX, Pdx-1^+^ cells were rare or non-existent in the control *Cdk4* ^WT^ pancreatic ductal epithelium (Fig. 5B). In contrast, higher numbers of ductal Pdx-1^+^:CK^+^ cells were observed in the *Cdk4* ^R/R^ pancreas before PX. The percentage of Pdx-1^+^:CK^+^ ductal cells increased at day 1 post-PX in the *Cdk4* ^WT^ pancreas and reached ∼3% at day 14 post-PX (Fig. 5B). This illustrates the capacity of normal pancreas to promote activation of Pdx-1^+^ cells in response to PX. Significantly, the activation was greater in the *Cdk4* ^R/R^ pancreatic ductal epithelium, where we observed a 5-fold increase in the ratio of Pdx-1^+^:CK^+^ cells within 1 day after PX, and a further significant increase in the ratio at day 2 post-PX. However, at day 14 post-PX, the ratio was reduced to a level comparable to the ratio observed in the *Cdk4* ^WT^ pancreas at day 14 post-PX. These observations demonstrate a significantly-increased pool of Pdx-1^+^:CK^+^ ductal epithelial cells in the regenerating *Cdk4* ^R/R^ pancreas as an immediate early response to PX.

### Increased Islet-Like Cell Clusters in *Cdk4* ^R/R^ Pancreas after Pancreatectomy

**Figure 6:**
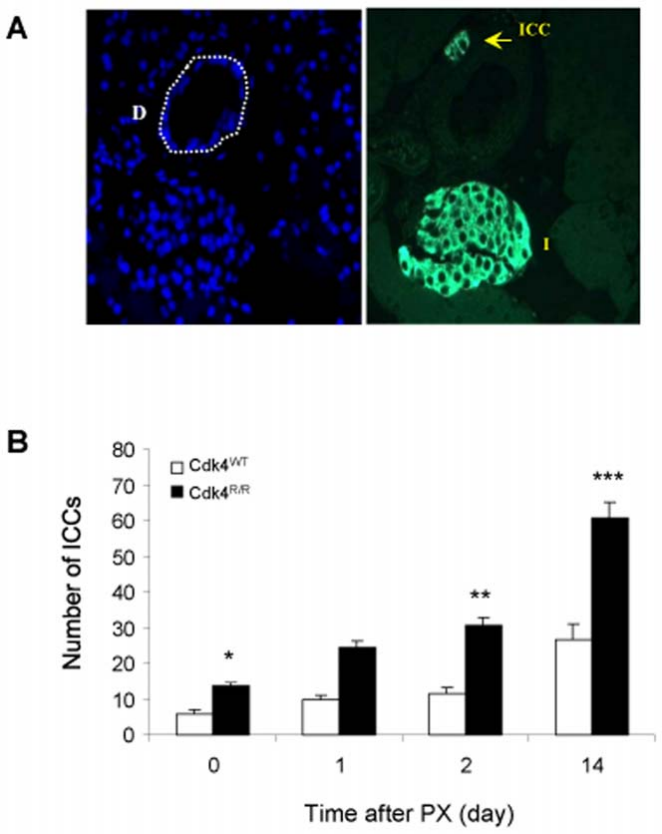
Increased number of islet-like cell clusters (ICCs) in pancreatectomized *Cdk4* ^R/R^ mice. (A) The number of ICCs that contain 5 or less insulin^+^ cells was counted. Left picture shows DAPI staining and the pancreatic duct (D) is identified by white stippled line. Right picture shows the mature islet (I) and an ICC (yellow arrow) identified upon immunofluorescence staining with insulin antibodies. (B) Number of ICCs was significantly increased before and after PX in *Cdk4* ^R/R^ mice (closed bars) compared with *Cdk4* ^WT^ mice (open bars). Values are the results obtained from four mice per group at each time point and expressed as mean number of ICCs per section. Error bars represent S.E. *p<0.05, **p<0.01, and ***p<0.001 *vs Cdk4* ^WT^ mice.

To determine whether insulin is expressed in the ductal epithelium of the *Cdk4* ^R/R^ pancreas, we performed immunofluorescence assays using insulin and CK antibodies to detect Ins and CK double positive cells. Ins^+^:CK^+^ cells were either rare or undetectable in the ductal epithelium of the *Cdk4* ^WT^ pancreas either before or after PX. In contrast, Ins^+^:CK^+^ cells were frequently seen in the ductal epithelium of the *Cdk4* ^R/R^ pancreas post-PX (data not shown). In response to regeneration, co-incident with the appearance of the rare Ins^+^ ductal epithelial cells, small islet-like cell clusters (ICCs) have been reported to originate in the vicinity of the ductal epithelium [49]. Further, ICCs are considered to represent newly-differentiated β-cells that originate from progenitors localized within the ductal epithelium. We next counted the number of ICCs (ie. those clusters with fewer than 5 β-cells) near the pancreatic ducts before and after PX in our experimental mice (Fig. 6A). Prior to PX, we observed a modest yet significant increase in the number of duct-associated ICCs in the *Cdk4* ^R/R^ pancreas (Fig. 6B). No increase in the number of ICCs was observed in the *Cdk4* ^WT^ pancreas by day 2 post-PX, although a significant increase in the number of ICCs was seen at day 14 post-PX. Interestingly, we detected a significant ∼3-fold increase in the number of ICCs in *Cdk4* ^R/R^ pancreas as early as day 2 post-PX (Fig. 6B). Moreover, the number of ICCs in the *Cdk4* ^R/R^ pancreas continued to rise significantly and by day 14 post-PX were increased by ∼5-fold compared with the number of ICCs prior to PX. Together, these observations demonstrate the ability of the *Cdk4* ^R/R^ pancreatic ductal epithelium to (i) proliferate extensively, (ii) express the putative pancreatic progenitor marker, Pdx-1, (iii) express insulin, a marker of mature differentiated β-cells, and (iv) promote the development of insulin-positive small ICCs.

## Discussion

**Figure 7:**
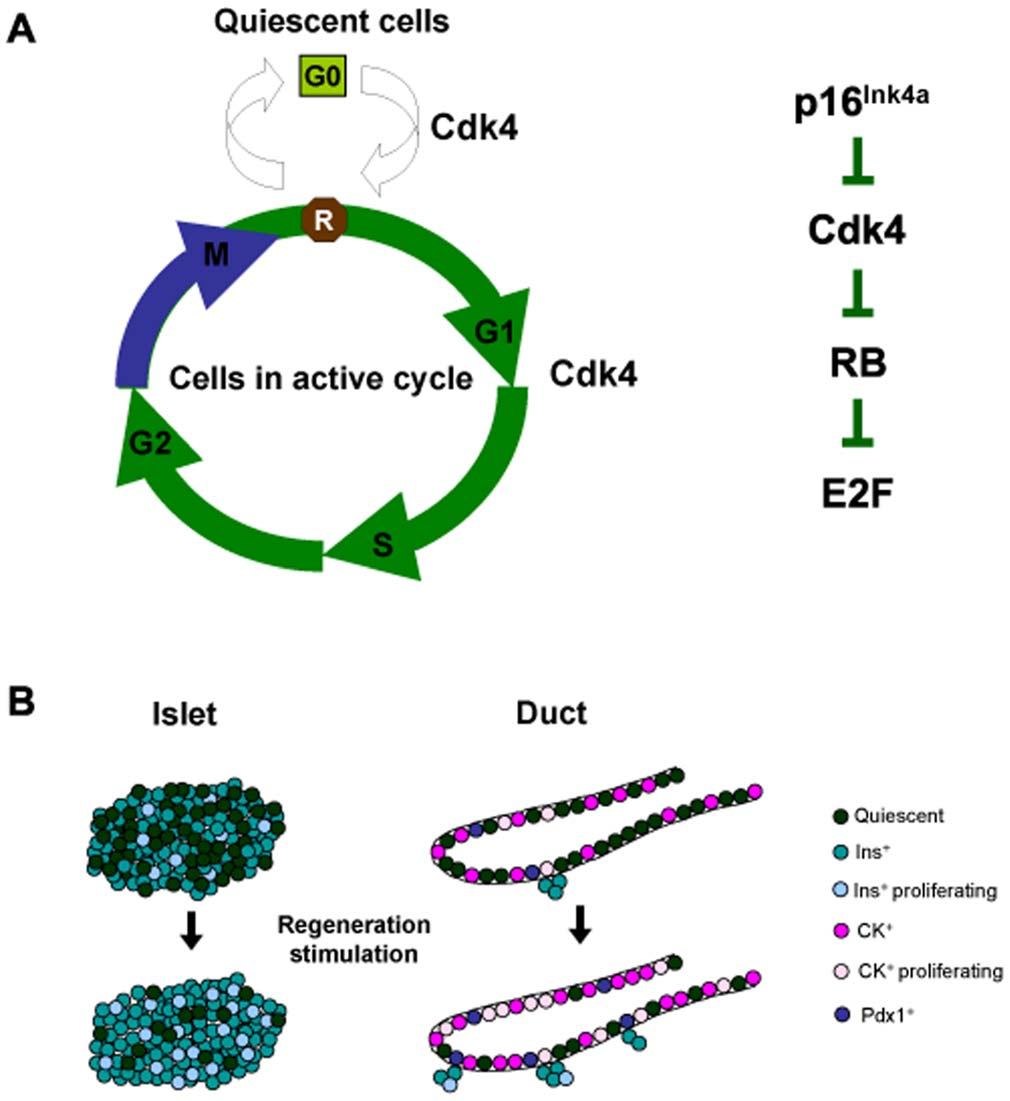
Proposed model of Cdk4-p16^Ink4a^-regulated regeneration of β-cell mass via effects on the islets and the pancreatic ductal epithelium. (A) Like all cells, β-cells and pancreatic ductal epithelial cells also traverse the cell cycle. A significant fraction of these cells are in the G0 state referred to as quiescence. Our data suggest that Cdk4 promotes cell cycle re-entry of quiescent cells. In addition, Cdk4 enhances cell cycle progression via stimulating G1 to S-phase transition. The p16^Ink4a^-Cdk4 pathway shown is operational during recruitment of quiescent cells into the cell cycle and to promote the G1 to S-phase cycling of cells already in cycle. These events are suppressed by p16^Ink4a^ and promoted by Cdk4 which inactivates RB thereby releasing E2F to drive S-phase progression. (B) Cdk4 appears to target the islets and the pancreatic ductal epithelium during a regenerative response to pancreatectomy and the proposed model outlines the events orchestrated by Cdk4. Islets are comprised of mature differentiated β-cells and a small fraction of these β-cells undergo proliferation in response to regeneration. Cdk4 promotes limited replication of pre-existing β-cells. In addition, we propose that majority of β-cells are quiescent. In response to pancreatectomy, Cdk4 recruits the quiescent cells into an active cell cycle, thereby increasing the pool of actively-dividing cells within the islet. Cdk4 appears to elicit a slightly different program in ducts in response to pancreatectomy. The duct is mainly comprised of cytokeratin-positive (CK+) epithelial cells, with rare proliferating cells, a limited number of cells that express the early pancreatic development marker, Pdx-1, and rare duct-associated ICCs. In response to pancreatectomy, the proliferation activity, Pdx-1 expression, and numbers of ICCs are increased by the cell cycle progression in addition to recruitment of quiescent ductal cells into the active cell cycle.

β-cells, like all other cells, are under the regulatory checks and balances enforced by changes in cell cycle progression [33]. Proof of this concept was provided using mouse models wherein the locus that codes for Cdk4, one of the important gate-keepers of the mammalian cell cycle machinery, was targeted by genetic recombineering [36], [53]. These studies illustrated that loss of Cdk4 results in β-cell hypoplasia and diabetes. In contrast, inheritance of a mutant Cdk4^R24C^ kinase, that is refractory to cell cycle inhibition by p16^Ink4a^, resulted in β-cell hyperplasia [35], [36]. Although these studies illustrated the unique role of Cdk4 in β-cell mass regulation, the mechanisms by which Cdk4 controls β-cell mass have remained unclear. Here, using a model of partial pancreatectomy, we showed that Cdk4 regulates post-natal β-cell mass by targeting two different cellular compartments: the pre-existing β-cells within islets and presumptive progenitors localized in the pancreatic ductal epithelium. Furthermore, we show that Cdk4 utilizes two distinct mechanisms to engineer β-cell mass re-constitution in response to pancreatectomy. First, the data is compatible with Cdk4 promotion of islet β-cell replication, a mechanism that is presumed to play a primary role in regulating β-cell mass. Second, Cdk4 catalyzes the recruitment of quiescent cells within the islets and the ductal epithelium to participate in the regenerative process. Interestingly, recruitment of quiescent cells within the ductal epithelium displays hallmarks of early pancreatic development [12]. Therefore, pancreatectomy induces rapid proliferation of ductal epithelial cells and a coincident significant increase in cells that express Pdx-1, a transcription factor that is involved in the earliest stages of embryonic pancreas development. The rapid proliferation kinetics combined with the appearance of Pdx-1^+^ cells suggests involvement of activated progenitors in response to injury. Moreover, we observe increased insulin-positive cells in the ductal epithelium and a significant enhancement in the number of duct-associated small ICCs. Together, we illustrate that Cdk4 stimulates β-cell regeneration by promoting β-cell replication and inducing progenitor cell activation by recruitment of quiescent cells into the active cell cycle (Fig. 7).

We estimated contributions of both the mechanisms - replication and recruitment from quiescence - to the overall increase in β-cell mass during 14 days after pancreatectomy (0.25±0.29 to 0.85±0.38 mg in *Cdk4* ^WT^ pancreas; and from 0.38±0.10 to 1.55±0.28 mg in *Cdk4* ^R/R^ pancreas). Estimated contributions by β-cell proliferation are 8% (∼0.02 mg) and 34% (∼0.13 mg) of the total increase of initial β-cell mass for *Cdk4* ^WT^ and *Cdk4* ^R/R^ pancreas, respectively (Fig. 4). Next, we estimated the ICC contributions that represent islet neogenesis from ducts. Assuming that 500 sections can be obtained from a pancreas and single cell mass can be estimated from water density (∼1.8×10^−6^ mg/cell), increasing numbers of ICCs correspond to 0.09 mg for *Cdk4* ^WT^ and 0.23 mg for *Cdk4* ^R/R^ pancreas. In spite of a large variation of β-cell mass changes, contributions from β-cell replication and ICCs (defined cell clusters including below 5 cells) are not sufficient to explain the β-cell mass increase during 14 days after pancreatectomy. Proliferation was assayed by two methods: (i) BrdU incorporation by intra-peritoneal injection, and (ii) by administration of CIdU and IdU via drinking water. Kushner's laboratory has shown that CldU/IdU labeling is as efficient as BrdU labeling at detecting β-cell proliferation [30], thus we believe that our results accurately reflect the numbers of proliferating cells in the Cdk4 models. However, it is possible that the BrDU and CIdU/IdU labeling indices may be under-estimated in our analyses although it is unlikely that would explain such a large gap in β-cell mass increase. Taken together, we suggest the possibility of non-proliferative contribution to the β-cell mass increase. For example, β-cell hypertrophy may be partially responsible for the observed β-cell mass increase. Further, mechanisms that involve compensatory de-differentiation could account for increase to β-cell mass. Indeed, Collombat and colleagues recently illustrated a mechanism of expansion of the β-cell mass in response to injury wherein pancreatic progenitor cells are capable of giving rise to glucagon-expressing α-cells that then transdifferentiate into β-cells [54], [55]. Importantly, it was shown that the continuous neogenesis of β-cells through the cell re-differentiation program did not involve β-cell self-renewal [54]. The possible existence of a large non-proliferative source of β-cells is surprising given the knowledge of replication-competent β-cells that drive regeneration of β-cell mass [28], [29], [30]. We do observe substantial numbers of proliferating cells in response to pancreatectomy and indeed Cdk4^R24C^ seems to promote the proliferation process. However, in addition we find a hitherto uncharacterized large non-proliferative pool of cells that Cdk4 can coax to participate in the regenerative response. It should be noted that distinct populations of quiescent and proliferative β-cells have been identified in another model organism recently [56].

Prior to the restriction point, cells can temporarily exit the cell cycle and undergo quiescence – a distinct out-of-cycle state that is under active genetic control (Fig. 7A). Previously, we and others have shown that loss of Cdk4 results in delayed S-phase entry from quiescence and a prolonged S phase [36], [53]. Here, we showed that Cdk4^R24C^ promoted β-cell proliferation within islets via a process of self-duplication. In addition, the mathematical model suggested that Cdk4^R24C^ may recruit quiescent cells in islets to re-enter the cell cycle. The most dramatic effect of Cdk4^R24C^ appears to be in the pancreatic ductal epithelium where we observed rapid proliferation activity and strong activation of the early developmental marker Pdx-1 within 24–48 hours after pancreatectomy. The appearance of Pdx-1^+^ cells in the regenerating ductal epithelium of the *Cdk4^R/R^* pancreas is suggestive of progenitor/stem cell activation in response to pancreatectomy and lineage tracing analyses will offer conclusive evidence in this regard. It has been postulated that the size of the pancreas is fixed during development due to the limited number of progenitor cells [57]. Our findings suggest that such presumptive progenitors may be responsive to context-dependent activation, such as injury. Similar activation of endocrine pancreatic progenitor cells, expressing the endocrine marker neurogenin 3 (Ngn3), has been reported in response to duct-ligation [31]. We did not observe Ngn3^+^ cells in our study before or after pancreatectomy. It is plausible that the two types of injuries, pancreatectomy versus duct-ligation, may foster activation of distinct progenitor cell populations that express either Pdx1 or Ngn3. Moreover, it is likely that the 60% pancreatectomy may be a relatively mild injury compared with the duct-ligation technique that may elicit a distinct genetic and physiological response. Alternatively, the kinetics of regeneration and the time-period of appearance of Ngn3^+^ cells in response to the two types of injuries are distinct enough to preclude detection of Ngn3^+^ cells in our model. Additionally, post-transcriptional regulation of *Ngn3* within the regenerating pancreas may preclude observation of Ngn3 positive cells as described elsewhere [58].

In summary, our findings suggest that the Cdk4-p16^Ink4a^ pathway regulates the long-term quiescent state of β-cells within islets and of potential progenitors within the pancreatic ductal epithelium (Fig. 7). Thus, reduced Cdk4 activity, due to an intact p16^Ink4a^ checkpoint, promotes an extended quiescent state, a property exhibited by a majority of β-cells. Activation of Cdk4, instead, overcomes the p16^Ink4a^ enforced quiescence and promotes re-entry of cells into the cell cycle. In addition, loss of the p16^Ink4a^ checkpoint may preclude already cycling cells from exiting the cell cycle into a quiescent state. Recruitment of quiescent cells to re-enter the cell cycle and promotion of cells within the cell cycle to continue dividing are central Cdk4-regulated mechanisms that control reconstitution of β-cell mass.

## Methods

### Generation of Mice

All experiments used age-matched (6–8 weeks old) female and male mice. All mice were housed in the animal facility at National Institutes of Health (NIH) in accordance with NIH regulations under specific pathogen-free conditions. All animal work is in accordance with the protocols approved by the NIH Animal Use and Care Committee.

### Partial Pancreatectomy

The entire splenic portion of the pancreas was surgically removed, resulting in a ∼60% pancreatectomy. Sham operation was performed as control for the multiple thymidine analog labeling experiment. Blood glucose level was determined by using an AscensiaELITE glucometer (Bayer Co., IN) before and after pancreatectomy.

### BrdU Labeling

Mice were given an intraperitoneal injection of 100 mg/Kg B.W. BrdU (Sigma, St. Louis, MO) 16 hr before sacrifice.

### Multiple Thymidine Analog Labeling

After partial pancreatectomy was performed, mice were continuously administered 5-chloro-2-deoxyuridine (CldU) or 5-iodo-2-deoxyuridine (IdU) in drinking water at 1 mg/ml for different time periods according to labeling scheme. CldU and BrdU were obtained from Sigma-Aldrich (St. Louis, Mo) and IdU was purchased from MP Biomedicals (Santa Ana, CA). During the chase period, the mice were given regular drinking water.

### Immunohistochemistry

Pancreatic tissue samples were harvested and fixed in 10% formalin solution. Paraffin-embedded sections (5 µm) were used in immunohistochemistry analyses. Sections were immunostained with guinea pig anti-insulin (Dako, Carpinteria, CA), mouse anti-pan cytokeratin (Dako, Carpinteria, CA), mouse anti-BrdU (Dako, Carpinteria, CA) or rabbit anti-Pdx-1 (Chemicon, Billerica, MA). For multiple thymidine analog labeling study, the staining procedure was according to the protocol published by Teta M. et al. (29). In brief, sections were first rehydrated, then microwaved in 0.01 M sodium citrate buffer, followed by permeabilization with 0.2% Triton X-100, incubation in 1.5 N HCL, and treatment with 0.25% trypsin (Invitrogen, Carlsbad, CA). Sections were then incubated to detect CldU using a rat anti-BrdU antisera (BU1/75; Accurate Chemical, Westbury, NY). IdU was detected using a mouse anti-BrdU antisera (BD Biosciences, Franklin Lakes, NJ). Guinea pig anti-insulin (Zymed Laboratories Inc., South San Francisco, CA) was used followed by incubation with secondary antisera conjugated to Cy2, Cy3 or Cy5 (Jackson ImmunoResearch Laboratories, West Grove, PA).

### Measurement of β-Cell Mass

For each pancreas, at least 3 inconsecutive sections with 150 µm separation were randomly chosen for staining. Sections were immunostained for insulin using a peroxidase indirect labeling technique. Quantitative evaluation of total β-cell mass was performed using image analysis software (Metamorph, CA). The areas of insulin-positive cells, as well as that of total pancreatic sections, were evaluated for each stained section. The relative volume of β-cells was determined by the stereological morphometry method, calculating the ratio between the area occupied by immunoreactive cells and that occupied by the total pancreatic tissue. Total β-cell mass per pancreas was derived by multiplying this ratio by the total pancreatic weight. Results represent the average from 4 animals per group.

### Proliferation Analysis

Stained pancreatic sections showed either insulin-positive β-cells or cytokeratin positive ductal cells together with BrdU-positive staining. At least 1000 β-cell nuclei or CK-positive ductal cells were counted per animal and at least 5 animals per group were subject to such analyses. The proportion of BrdU-positive β-cells (or BrdU-positive ductal cells) to total number of β-cells (or total number of ductal cells) was quantified. The result represents the percentage of proliferating rate in either β-cells or ductal cells.

### Islet-Like Cell Clusters (ICCs)

The number of islet-like cell clusters that comprise five or less β-cells was counted subsequent to insulin immunohistochemistry.

### Mathematical Modeling

We developed a mathematical model to examine the characteristics of cell proliferation from the labeling results of CldU^+^, IdU^+^, and CldU^+^:IdU^+^ cells (Fig. 3A). To distinguish the different potential for cell proliferation, we separated cell populations into quiescent and active cells. We denote the number of quiescent cells with 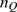 and the number of active cells with 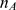. Quiescent cells do not proliferate:
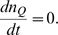



On the other hand, active cells proliferate with a given time-dependent proliferation rate 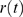. When active cells proliferate, mother and daughter cells become labeled with thymidine analogs in newly-synthesized DNA after mitosis. Note that we assumed symmetric DNA segregation. Therefore, a proliferated active cell becomes two labeled cells:
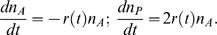



Here, we neglect multiple proliferations of once-proliferated cells because the event is sufficiently rare (Table S1). The rate of cell proliferation 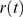 may begin to increase by the PX stimulation, and diminish after a certain time point. Therefore, we use the following functional form multiplying two sigmoidal functions for rising and falling phases:
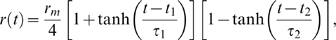
where 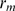 is the magnitude of the proliferation rate; 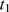 and 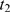 represent the time points for rising and falling phases at which the proliferation rate has the half maximal value of 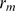; finally, 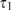 and 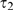 give the time scales for the slopes of the rising and falling phases, respectively. The functional form is general enough to describe even monotonically-increasing proliferation rate during a measuring period, if 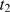 is larger than the given period. We apply this basic model to examine cell proliferation. For a time period from an initial time 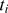 to a final time 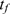, the ratio of labeled cells to unlabeled cells is
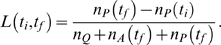



For example, in the 2-2-4 labeling protocol, the CldU^+^ labeling ratio becomes 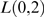; and the IdU^+^ labeling ratio becomes 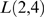. In case of single labeling with BrdU, the labeling ratio from day 1 to day 2 becomes 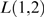. Note that we use 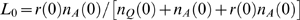 for calculating basal labeling ratio per day.

### Optimization

We have two kinds of cell proliferation information. First, single labeling with BrdU represents cell proliferation at day 0, 1, 2, and 14. Second, double labeling with CldU and IdU represents accumulated cell proliferation for a given period that depends on the experimental protocols: 2-2-4, 2-2-14, and 7-7-14 (Fig. 3B). Therefore, we have totally ten sets of independent labeling data (four with BrdU, three with CldU, and three with IdU): 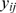 with 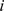 = 1, 2, …, 10 for each ensemble of 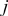 = 1, 2, …, 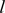. Using the mathematical model, we examine characteristics of cell proliferation which can appropriately explain the results of both experiments. In the labeling results, scale of labeling ratio is not comparable between BrdU and CldU/IdU labeling; BrdU could detect more proliferated cells. The difference may result from the injection method difference (intraperitoneal injection of BrdU vs. by drinking water that includes CldU/IdU). It is also possible that we observed CldU/IdU-labeled cells in lower laser intensity and less laser-exposure time. Therefore, we introduce a scale parameter η to scale up CldU/IdU labeling results; 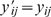 for 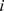 = 1, …, 4 and 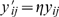 for 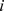 = 5, …, 10. To compare these 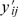 with model predictions 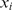, we define a cost function, quantifying the deviation between them,
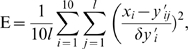
where we consider the experimental uncertainty 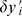 as a weight; as the error 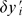 is smaller, we weight the corresponding data 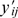 as more significant. The uncertainty is the standard deviation of data 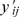 for the 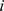
th labeling ratio:
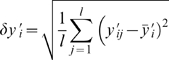
with the mean value 
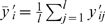
.

Then, we simulate the model with the parallel tempering Monte-Carlo (MC) method (60) for sampling in the seven-dimensional parameter space, 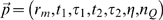, and find the best parameter 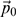 minimizing the cost 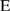. To obtain the global minimum of the cost function, the MC method simultaneously runs several chains with different temperatures (fluctuations). It allows both coarse and fine search for the best parameter set. In particular, we used ten temperatures from 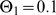 to 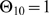 with a uniform spacing interval of 0.1. The updating probability of the MC simulation is 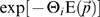 for the 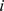
th chain. In addition, after every 20 time-steps, we randomly picked two chains of different temperatures (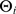 and 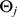) and exchanged their parameter set (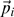 and 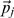) with the probability 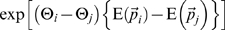 to find the global minimum of the cost function more efficiently. After finding the best parameter set, we ran 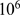 MC steps with one fixed temperature (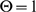) from the best parameter set found in the equilibration. From the random walk of 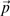 around 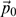, we can estimate the average and the standard deviation of optimal parameter values. At first, we found the most probable values of the scale parameter η (2.4±0.9 and 2.3±0.8 for of β-cells in Cdk4^WT^ and Cdk4^R/R^ mice, respectively; 2.2±0.9 and 3.7±0.2 for of ductal cells in Cdk4^WT^ and Cdk4^R/R^ mice, respectively). Therefore, as a universal scale parameter that should consistently apply all the CldU/IdU labeling experiment, we fixed the scale parameter with η = 3; then, we treated η as a constant, not a parameter. For the model comparison, we used the Bayesian model probability 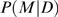 that estimates how a model can describe a given data well [59]. The probability can be calculated from the MC simulation with different temperatures: for using 10 chanis, 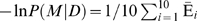. Here, 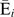 means the average cost for each temperature 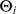. Note that for this calculation, there is no exchange between MC chains of different temperatures in contrast to the previous procedure for searching a global minimum of a cost function. In addition, we used 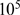 MC steps to determine the model probability 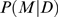.

### Statistical Analysis

All results are reported as means ± SE. Statistical analysis was performed by independent t-test (unpaired and two-tailed).

## Supporting Information

Figure S1 (2.90 MB TIF)

Figure S2 (6.84 MB TIF)

Figure S3 Model probabilities of population ratio of quiescent to active cells for β-cells and ductal epithelial cells of *Cdk4^WT^* and *Cdk4^R/R^* mice.
(0.86 MB TIF)

Table S1 (0.05 MB DOC)
