# Supplementary figures and images for: Cdk4 Regulates Recruitment of Quiescent β-Cells and Ductal Epithelial Progenitors to Reconstitute β-Cell Mass
Source: PLoS One. 2010 Jan 13;5(1):e8653. doi: 10.1371/journal.pone.0008653 (PMC2801612; doi:10.1371/journal.pone.0008653)

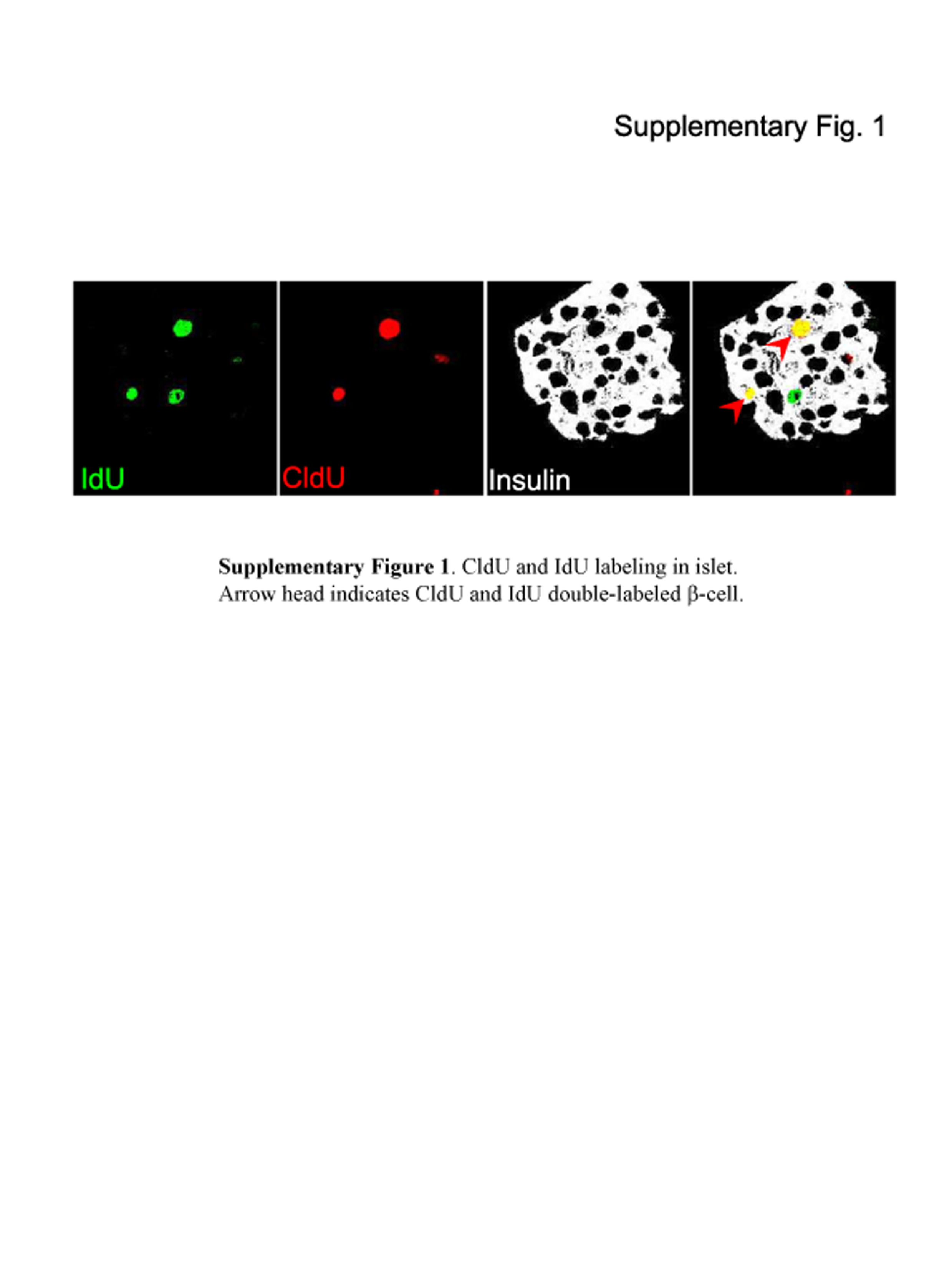

Supplement: Figure S1 — (2.90 MB TIF) [file pone.0008653.s001.tif]

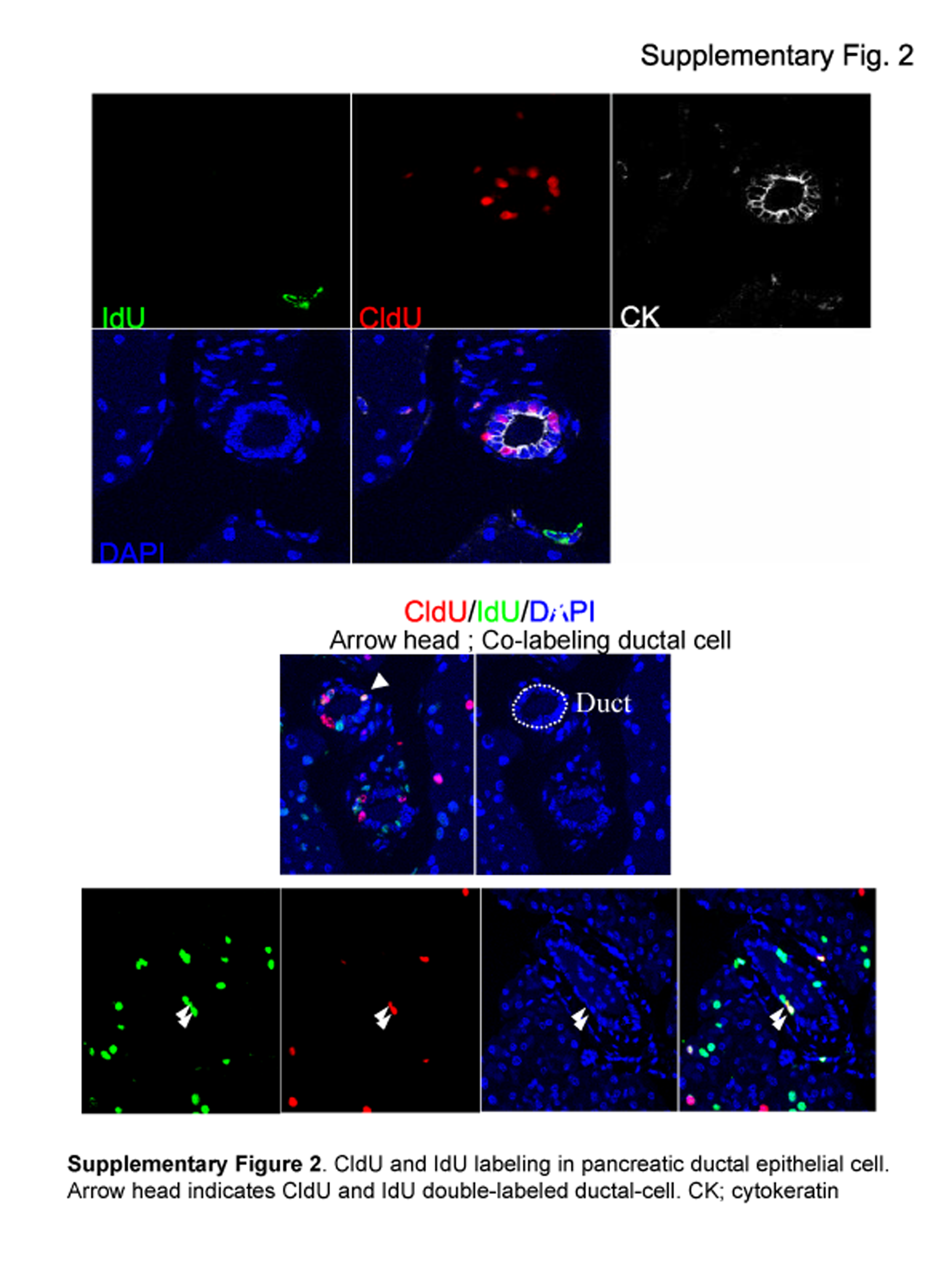

Supplement: Figure S2 — (6.84 MB TIF) [file pone.0008653.s002.tif]

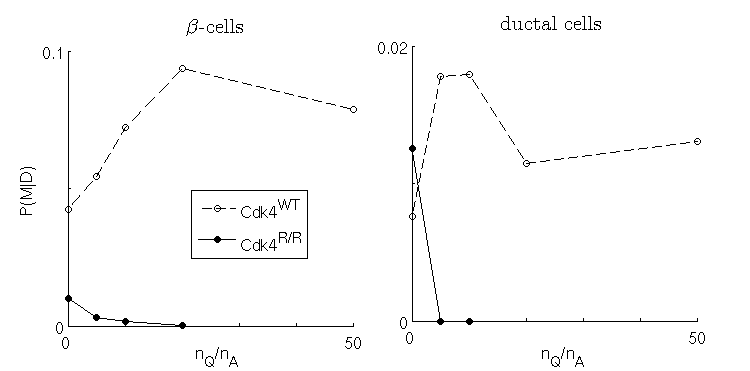

Supplement: Figure S3 — Model probabilities of population ratio of quiescent to active cells for β-cells and ductal epithelial cells of Cdk4WT and Cdk4R/R mice. (0.86 MB TIF) [file pone.0008653.s003.tif]
