# Supplementary material for: Cdk4 Regulates Recruitment of Quiescent β-Cells and Ductal Epithelial Progenitors to Reconstitute β-Cell Mass
Source: PLoS One. 2010 Jan 13;5(1):e8653. doi: 10.1371/journal.pone.0008653 (PMC2801612; doi:10.1371/journal.pone.0008653)
Supplement: Table S1 — (0.05 MB DOC) [file pone.0008653.s004.doc]

**Supplementary Table 1. Labeled cell numbers.**

| Protocol | Labeling | *Cdk4*^WT^ β-cells | *Cdk4*^R/R^ β-cells | *Cdk4*^WT^ duct cells | *Cdk4*^R/R^ duct cells |
| --- | --- | --- | --- | --- | --- |
| 2-2-4 | CldU | 47/2377 | 35/3440 | 60/2643 | 37/1676 |
|  | IdU | 105/2377 | 111/3440 | 102/2643 | 280/2676 |
|  | CldU+IdU | 8/2377 | 3/3440 | 6/2643 | 8/1676 |
| 2-2-14 | CldU | 22/2229 | 93/3326 | 26/1474 | 54/1328 |
|  | IdU | 45/2229 | 193/3326 | 27/1474 | 40/1328 |
|  | CldU+IdU | 3/2229 | 10/3326 | 0/1474 | 6/1328 |
| 7-7-14 | CldU | 38/1716 | 235/4512 | 143/4031 | 280/4284 |
|  | IdU | 38/1716 | 402/4512 | 337/4031 | 112/4284 |
|  | CldU+IdU | 0/1716 | 8/4512 | 26/4031 | 13/4284 |

**Note that numbers are represented as corresponding labeled cells per total counted cells.**
